# BAMSI: a multi-cloud service for scalable distributed filtering of massive genome data

**DOI:** 10.1186/s12859-018-2241-z

**Published:** 2018-06-26

**Authors:** Kristiina Ausmees, Aji John, Salman Z. Toor, Andreas Hellander, Carl Nettelblad

**Affiliations:** 1 Department of Information Technology, Uppsala University, Box 377, Uppsala, Sweden; 2 Department of Biology, University of Washington, Box 351800, Seattle, 98195 USA

**Keywords:** Human genome, 1000 genomes, Big data, Next-generation sequencing, Cloud computing

## Abstract

**Background:**

The advent of next-generation sequencing (NGS) has made whole-genome sequencing of cohorts of individuals a reality. Primary datasets of raw or aligned reads of this sort can get very large. For scientific questions where curated called variants are not sufficient, the sheer size of the datasets makes analysis prohibitively expensive. In order to make re-analysis of such data feasible without the need to have access to a large-scale computing facility, we have developed a highly scalable, storage-agnostic framework, an associated API and an easy-to-use web user interface to execute custom filters on large genomic datasets.

**Results:**

We present BAMSI, a Software as-a Service (SaaS) solution for filtering of the 1000 Genomes phase 3 set of aligned reads, with the possibility of extension and customization to other sets of files. Unique to our solution is the capability of simultaneously utilizing many different mirrors of the data to increase the speed of the analysis. In particular, if the data is available in private or public clouds – an increasingly common scenario for both academic and commercial cloud providers – our framework allows for seamless deployment of filtering workers close to data. We show results indicating that such a setup improves the horizontal scalability of the system, and present a possible use case of the framework by performing an analysis of structural variation in the 1000 Genomes data set.

**Conclusions:**

BAMSI constitutes a framework for efficient filtering of large genomic data sets that is flexible in the use of compute as well as storage resources. The data resulting from the filter is assumed to be greatly reduced in size, and can easily be downloaded or routed into e.g. a Hadoop cluster for subsequent interactive analysis using Hive, Spark or similar tools. In this respect, our framework also suggests a general model for making very large datasets of high scientific value more accessible by offering the possibility for organizations to share the cost of hosting data on hot storage, without compromising the scalability of downstream analysis.

**Electronic supplementary material:**

The online version of this article (10.1186/s12859-018-2241-z) contains supplementary material, which is available to authorized users.

## Background

The 1000 Genomes project has produced one of the world’s largest public collections of sequenced human genome data with the goal of providing a public resource giving a wide representation of human genetic variation [1]. This data is useful for many applications, including the investigation of genomic causes of diseases. For many applications, curated released variant files may be sufficient. However, for more specialized questions such as validation of specific candidate mutations or screening for variants with incomplete calling performance, it can be necessary to use the aligned sequencing reads. The alignment data released by the 1000 Genomes project is made available in the BAM (Binary Alignment/Map) format. BAM is the binary version of the SAM (Sequence Alignment/Map) format used by the SAMtools software [2], and it is the expected primary format of aligned data received from mature sequencing platform pipelines. For each BAM file, there are two auxiliary files containing indexing and statistics. For all 2535 individuals taken together, the resulting size of the data is in total roughly 60 TB, with one BAM file containing all aligned data per individual. The data is available in its entirety from a number of mirrors, in addition to the authoritative original source.

Mature open source software to analyse and work with individual BAM files, most prominently SAMtools itself, are readily available, but the sheer size of the complete dataset makes analysis expensive, and calls for scalable distributed computing solutions. In that category, HadoopBAM [3] based on Hadoop/MapReduce [4] or ADAM [5] based on Apache Spark [6] have been demonstrated to accelerate the processing of large volumes of genome data, but the adoption of these tools comes with a steep learning curve for end-users without distributed computing experience. Furthermore, such frameworks work best in the scenario when the entire dataset is available in a resilient, compatible datastore such as the Hadoop Distributed File System (HDFS) [7], meaning that data has to be staged into the system prior to the computations. However, for many organizations, the cost and complexity in maintaining a dedicated system for Big Data processing, and the cost of storing a local copy of the entire dataset, is substantial. Still, such an approach can make sense for applications requiring frequent access to the original data, such as iterative processing. It can also make sense for complex ad-hoc analysis requiring the full flexibility of e.g. Apache Spark. For simpler filtering tasks, however, it introduces an unnecessary level of complexity.

The availability of private and community cloud computing infrastructure is a rapidly rising trend in the academic e-infrastructure landscape. Infrastructure as-a Service (IaaS) clouds complement traditional HPC batch resources by offering the flexibility to rapidly deploy analysis environments on demand. One such example is the Swedish National Infrastructure for Computing (SNIC) Science Cloud (SSC) [8], a national cloud resource built on OpenStack [9]. SSC offers virtual compute and storage resources closely co-located with traditional HPC clusters and shared storage pools. SSC participates in Glenna2, comprising similar initiatives in the Scandinavian countries. On a European level, the European Open Science Cloud initiative can be expected to accelerate the adoption of private and hybrid cloud infrastructure. Significant efforts are already made in that direction with the EGI Federated Cloud (FedCloud) initiative [10], and HelixNebula – The Science cloud [11], a EUR 5.3M pre-commercial tender to establish a hybrid cloud platform. The Open Science Data Cloud (OSDC) [12] is a large initiative to provide large datasets of high scientific value closely located with OpenStack cloud computing resources for flexible and efficient analysis. For the Bioinformatics community, the ELIXIR Embassy Cloud [13] provides OpenStack resources co-located with EMBL-EBI’s data resources.

The main goal of the work in this paper is to develop a modern, scalable solution for massive filtering of genome data, capable of leveraging this emerging cloud infrastructure landscape. To that end, we propose a solution and associated cloud service framework, the BAM Search Infrastructure (BAMSI), for filtering of massive genome data that avoids the issues of duplicity and storage limitations. BAMSI is capable of leveraging data from several distinct locations to provide an efficient distributed tool for filtering and analysing the raw dataset. We allow multi-cloud configurations by being able to spawn and use computational resources close to data in OpenStack-based [9] clouds as well as other IaaS providers such as Amazon EC2. In Sweden, a mirror of the 1000 Genomes dataset is available on shared storage at the Uppsala Multidisciplinary Center for Advanced Computational Science (UPPMAX). Similarly, the dataset is publicly available in the Amazon S3 public cloud storage free of charge [14]. With data available close to cloud compute infrastructure (in our example SSC and Amazon EC2) BAMSI moves computations close to data provisioning of local, transient virtual compute nodes close to the data source. This model minimizes network bottlenecks and increases filtering throughput.

In this paper we introduce a publicly available deployment of BAMSI, and present an analysis of the performance and scalability of the framework, illustrating the benefits of such a multi-cloud configuration. We envision our service to be useful together with a diverse set of downstream analytics platforms such as Hadoop (Pig, Hive) [15, 16], Spark and ADAM, since we offer a method to pre-filter the dataset, greatly reducing the amount of data that needs to be staged into those environments. For highly compressive filters, the resulting subsets can also be downloaded locally and further analysed with a range of conventional bioinformatics tools or statistical computing platforms such as R and Python.

To illustrate the potential of BAMSI, we also present a rudimentary structural variant analysis on the entire 1000 Genomes phase 3 set of aligned reads. First, we use BAMSI to execute a whole-genome filter for alignments where the paired-end reads map to locations in the reference genome that would indicate a total template length exceeding 600 base pairs. Since this is inconsistent with the fragment generation protocols, such reads are indicative of deletion/inversion events moving the paired sequences closer, or alignment errors. We then perform additional filtering on this reduced data set in order to isolate inversion events, and produce a genome-wide overview of potential regions with high inversion frequency.

## Implementation

### System overview

Three main objectives have been driving the development of the framework. First, use of the service should be intuitive and accessible for a scientist with no experience of distributed computing. Second, the entire dataset should not have to be stored on the analysis platform. Finally, the framework should be capable of making simultaneous use of multiple mirrors of the data, and it should be capable of moving filtering workers close to data to increase the throughput of the analysis.

**Fig. 1:**
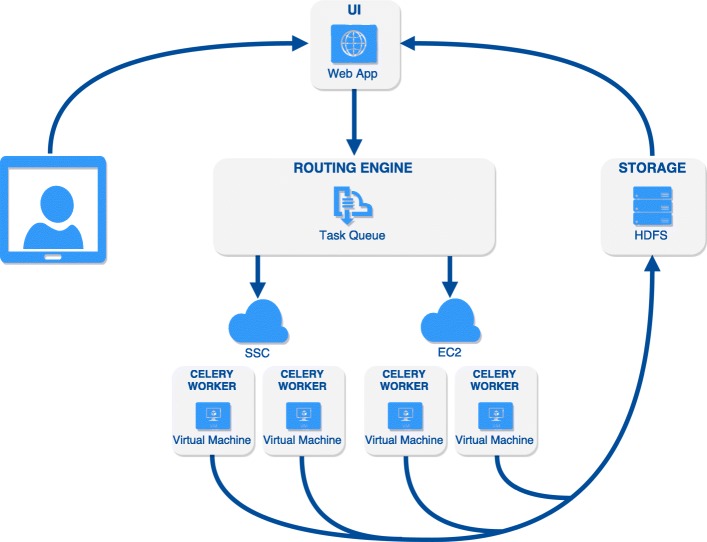
Overview of the architecture. The user defines a data filtering job in a graphical user interface or using a REST API. The routing engine distributes tasks to workers residing in one or several cloud platforms, each with a configured source of the data. The filtered results can be routed to a permanent or transient storage location (such as an HDFS cluster) for further downstream analysis with other tools, or for download via the interface

Figure 1 illustrates the design of the framework. Four components are central to the system: the User Interface (UI), the Routing Engine (RE), the Worker Ecosystem (WE) and the Storage Repository (SR). The user interacts with BAMSI via the UI, which allows filter jobs to be defined, launched, and monitored. A job consists of a filter condition and the set of files to apply it to. When a job is launched, separate tasks are defined and created, with each task corresponding to one BAM file to filter. The tasks are dispatched to available worker resources by the RE. The WE comprises all compute resources, or workers, configured to execute filter tasks. Finally, the output from all workers is consolidated to the SR, from which the user can access the reduced dataset for further analysis. Below, we briefly touch on the main layers to describe their design and interaction.

### User interface (UI)

**Fig. 2:**
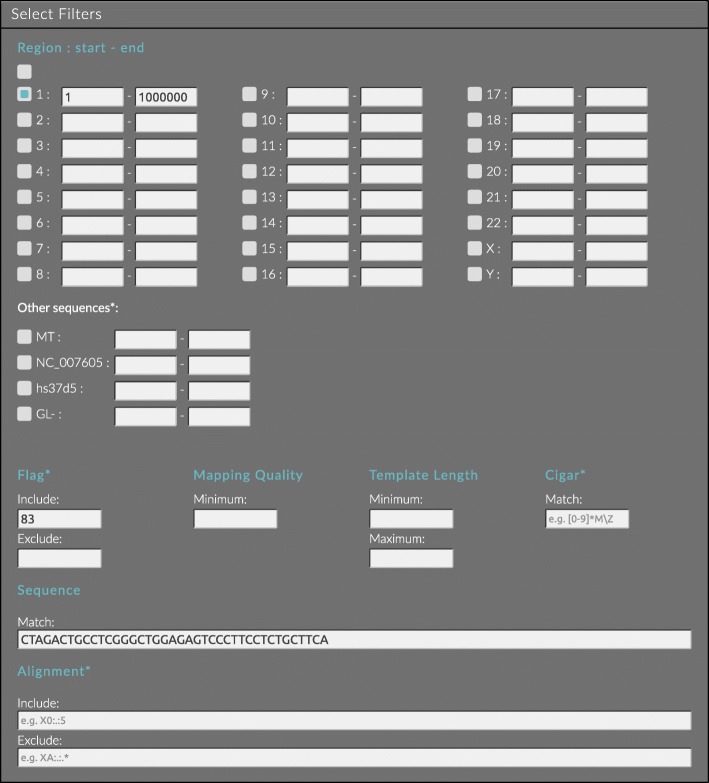
Example of specifying a filter query to select all alignments from the first Mb of chromosome 1, with the sequence containing a given pattern of nucleotides. The flag 83 is also required, meaning that the alignments should have flag bits 0x1, 0x2, 0x10 and 0x40 set, corresponding to a paired read, mapped in a proper pair to the reverse strand, and being the first segment in the template

The BAMSI UI allows the user to specify and deploy filter queries, view the status of the system, monitor progress of tasks and to download the resulting filtered-out data. Users familiar with SAMtools will recognize the standard filter options such as minimum mapping quality and flag bits to include or exclude. In addition to these, it is also possible to specify a range for the template length, a pattern of nucleotides that the sequence must contain, constraints on the format of the cigar string, and criteria on the tags in the optional alignment field of the BAM records. The latter two are specified using regular expressions that the value found in the BAM record is matched against. Subsets of the original set of files can be selected by specifying the populations, individuals and genomic regions that are of interest. Figure 2 shows a screenshot of a typical filtering configuration; alignments from the first Mb of chromosome 1 containing a given sequence of nucleotides, and having flag bits set that indicate a paired read, mapped in a proper pair to the reverse strand, and being the first segment in the template.

Submitted jobs are given a tracking id by which the user can monitor progress via the dashboard page. Statistics of the job’s progress are displayed, as well as a searchable table containing details of each task, allowing finished ones to be downloaded via the browser. The output format of the filtered dataset is selected at query deployment. Supported formats are BAM and individual alignment format; a modification of the SAM format that excludes the header and includes the individual and region information in every alignment. The alignments thus become self-contained units, rendering the data suitable for imposing structure and performing interactive analysis using a query language, or for processing within a distributed computing framework such as Hadoop.

### Routing engine (RE)

The RE handles the dispatch of tasks and maintains handles to monitor their progress. BAMSI exploits the Celery [17] messaging and queuing fabric to disseminate tasks across workers. A simple configuration with one queue and distribution of tasks to workers as they become available is currently implemented.

### Worker ecosystem (WE)

The WE is automatically managed by the Celery framework. As resources hosting the service are spawned, they join the global pool of workers via a queue and become available to receive filter tasks. Environment-specific settings such as IP addresses and ports for communication are defined using a configuration file, where the mirror of the data is also specified by means of a file path (e.g. a mounted directory on the system where the worker is running, or a HTTP or FTP URL). The worker logic is implemented as a wrapper and extension of SAMtools; when a task is received, the specified BAM file is streamed from the configured data source and filtered according to the given condition. The resulting data is finally pushed to the SR and the worker is ready to receive another task.

### Storage repository (SR)

The storage backend of BAMSI is designed to be pluggable and adaptable. Users setting up their own instance of BAMSI can configure a storage repository of choice, ideally on the provider where subsequent analysis of the data will be performed. The design is adapted to any system that supports REST interface, so providers such as Swift, S3 and Microsoft Document Cloud would be compatible. The publicly available deployment of BAMSI implements HDFS as storage repository.

### Python API

**Fig. 3:**
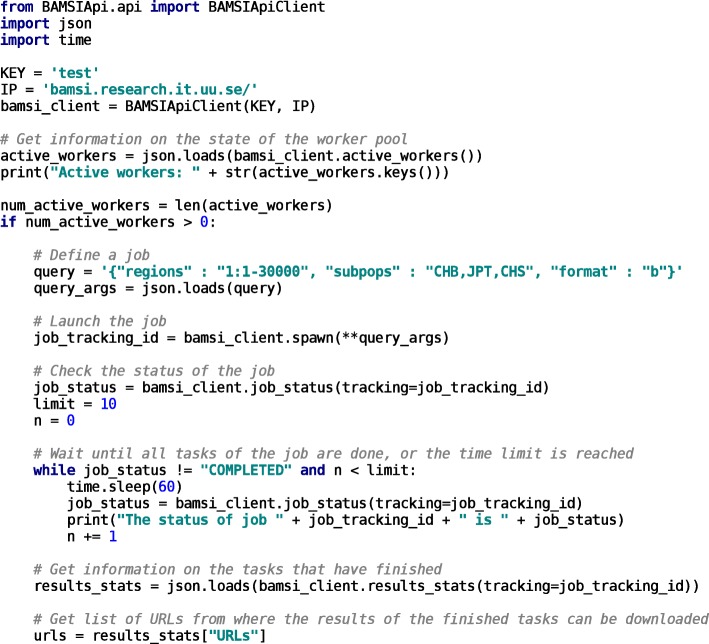
Example of interaction with BAMSI via the Python API. First, the state of the worker pool is probed. If there are any active workers, a job to filter out alignments from the first 30000 bp of chromosome 1, in individuals from three subpopulations, is defined and launched. The status of the tasks is probed until all are finished, or a time limit is reached. Finally, a list of URLs from which the results of the finished tasks can be downloaded is fetched

A Python API is also provided as an alternative to the UI. It supports the same functionality for interaction with BAMSI as the graphical interface, including the deployment and monitoring of tasks and viewing the state of the worker pool. Figure 3 shows an example of using the API to launch a task, monitor its progress, and get a list of URLs from which the results can be downloaded. The API is available at https://github.com/NGDSG/BAMSI-API.

## Results

To demonstrate the utility of BAMSI, we evaluate the performance benefits of the multi-cloud setup, and present a possible use case of the framework. The performance was evaluated in terms of aggregated filtering throughput. For a particular BAMSI setup and deployment, the throughput will depend on a number of factors, including computational efficiency, network speeds and write performance of the SR. The user can increase throughput by adding workers to the WE, but since the horizontal scaling is limited by the eventual saturation of the link to the data backend, we focused on investigating how the use of multiple data sources affects scaling. As a case study, we chose to perform a structural variation analysis on the entire 1000 Genomes phase 3 low-coverage data data set. We used BAMSI to scan the data for alignments indicative of possible inversion events, and present a genome-wide overview of the results.

### Horizontally scalable filtering using a multi-cloud BAMSI deployment

To illustrate the capability of BAMSI to increase throughput by aggregating multiple mirrors of the data, a deployment with workers in two different cloud backends was configured. One set of resources was deployed on SSC, using the mirror of the 1000 Genomes data available on UPPMAX. Each such virtual machine had 2 VCPUs, 40GB disk, 4GB RAM. The second set was deployed on Amazon EC2, accessing the data from the publicly available Amazon S3 bucket. The EC2 resources had 2 VCPUs, 8GB disk, 8GB RAM (m4.large). One celery worker was deployed per machine, with concurrencies 6 and 4 in the SSC and EC2 instances, respectively. Concurrency determines the number of threads running in the worker. As the optimum depends on several factors, a pre-analysis was performed to find suitable values.

For a given query, the throughput is defined as the total disk size of streamed and filtered data per unit of runtime, measured as the wall time from query deployment to the completion of the last task. As an indicator of the efficiency of the system when given additional workers, we also report the scaled speedup, defined as $\frac {Speedup_{n}}{n}$ where *n* is the number of workers and the speedup is defined as the ratio of runtime using 1 worker to the runtime on *n* workers: $Speedup_{n} = \frac {T_{1}}{T_{n}}$. The query used for performance analysis was to select all alignments with a minimum observed template length (as reported by the field TLEN in the BAM file) of 600 bp from a set of 520 files with a total disk size of 11717 GB. The output format was set to BAM, and due to varying latency of access to the SR for different compute providers, filtered-out data was written to local disk only.

Three suites of tests were performed, the first of which only used the compute resources deployed on SSC. Throughput was measured when running the query using varying numbers of celery workers. In the second suite, the additional EC2 compute resources were included. For this set of runs the number of SSC machines was fixed at 12, and throughput was measured for varying numbers of additional workers on EC2. The third suite was performed using EC2 resources only, in order to put the results for the multi-cloud setup into context, and illustrate the baseline performance of EC2. Since all configurations utilized shared resources with varying performance, the query was run three times per setup. We report the maximum throughput over these runs.

**Fig. 4:**
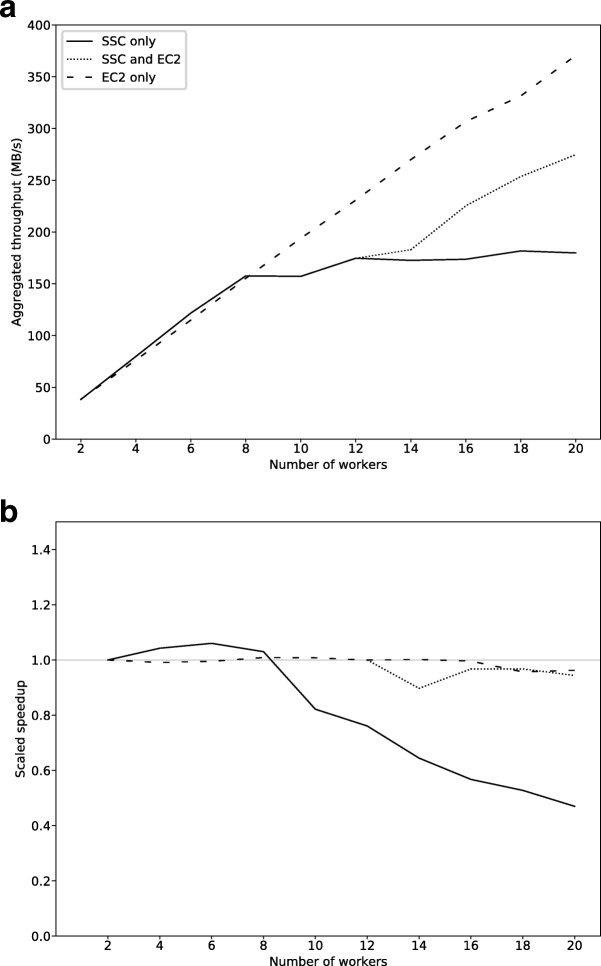
Performance evaluation results. **a** Total throughput as a function of number of celery workers. The solid line indicates runs in which all workers were deployed on SSC. The dotted line indicates runs in which the number of SSC workers was fixed to 12, with additional workers deployed on Amazon EC2. The dashed line indicates runs in which workers were deployed on Amazon EC2 only. The maximum throughput over three runs is plotted for each setup of celery workers. **b** Scaled speedup for the same experiments as above. The gray line indicates a scaled speedup of 1, corresponding to linear scaling

The resulting throughputs are displayed in Fig. 4a. The solid line indicates the runs in which only SSC resources were used, with a leveling-out of throughput occurring around 170 MB/s at 12 workers. Saturation of the link to UPPMAX was reached at this point; adding workers no longer increased throughput. With additional workers instead being added on EC2 from the point of saturation, throughput continued increasing further, as indicated by the dotted line. The dashed line shows the performance of using EC2 only. As expected, saturation of the S3 data source was not reached. Figure 4b shows the performance in terms of scaled speedup. The theoretical upper bound for this metric is 1.0, which corresponds to linear speedup; the system performing twice as fast when the number of workers is doubled. The fact that superlinear speedup is reached for the SSC only runs can be explained by varying performance due to running on shared resources. Comparing the scaled speedup for the two scenarios in which EC2 workers were used (dotted and dashed lines) shows similar behavior, indicating that there was no significant overhead of adding EC2 resources on top of the BAMSI deployment on SSC, as opposed to running on EC2 only.

### Using BAMSI for structural variation analysis

For the structural variation analysis, BAMSI was used to perform an initial filtering of the entire data set on the condition of a minimum observed template length of 600 bp. The results were stored in HDFS in the independent alignment format, where the distributed processing framework Hive was used for subsequent filtering. The Hive queries used can be found in Additional file 1.

**Fig. 5:**
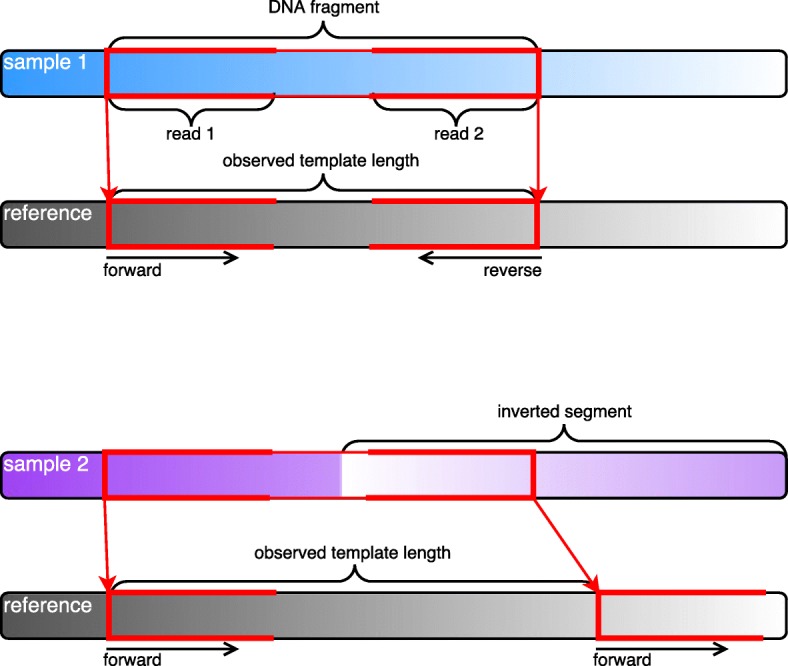
Schematic representation of alignment of paired-end reads to a reference sequence. Sample 1 has no structural variation w.r.t. the reference; read 1 aligns to the forward strand and read 2 to the reverse. Sample 2 has an inversion with respect to the reference, giving rise to two reads with forward orientation. In order to isolate potential inversion events, we kept kept such alignments, as well as the analogous case with two reverse-mapped reads, by requiring that both reads in an alignment have the same orientation as indicated by the SAM flag 0×10

To isolate potential inversion events, only alignments in which both reads mapped to the same strand were kept. This was done by enforcing that both reads in each pair had the same orientation as indicated by the SAM flag 0×10. Figure 5 shows a schematic representation of how this type of structural variation is expressed in paired-end sequencing. Sample 1 shows the typical case with no structural variation w.r.t. the reference; read 1 aligns to the forward strand and read 2 to the reverse. The DNA sequence of sample 2, however, has an inversion with respect to the reference, causing read 2 to be mapped in the opposite direction, resulting in both reads having the same direction in the alignment. This type of alignment also gives rise to an observed template length that is larger than the fragment size of the sequencing protocol, motivating the filter of minimum template length 600 bp as an initial data-reduction step. The case with two reverse-aligned reads is analogous. In order to reduce noise, cases with alternative alignments or at least one read that did not completely match the reference were discarded. This included discarding alignments with reads that did not have a cigar string on the form nnM or contained any XA-tags. We further required that every alignment should have at least 20 supporting alignments from distinct individuals. This was done by projecting the start positions and template lengths of each alignment down to kb scale, counting the number of distinct individuals in each such bin, and only selecting alignments that were in bins with an individual count of at least 20.

The results are presented as a low-resolution heat map of each chromosome in order to give an overview of areas of potential interest. Starting position in each chromosome, projected to Mb scale, is given on the y-axis and observed template length projected to 10 kb scale on the x-axis, with intensity representing the frequency of unique individuals having an alignment in each such bin.

**Fig. 6:**
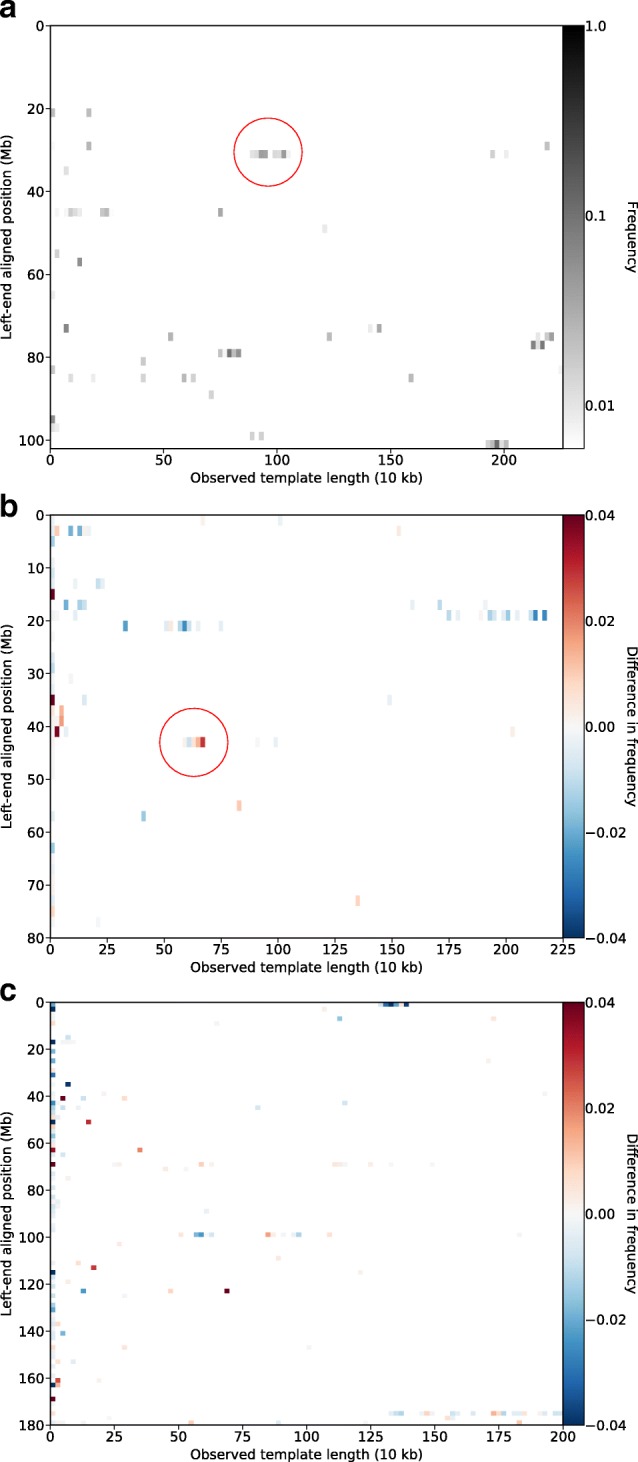
Chromosome-wide heat maps of potential inversion alignments found in the 1000 Genomes phase 3 data set, with start position plotted against observed template length. **a** Alignments found in chromosome 15, from the entire set of 2535 individuals. The fraction of individuals having an alignment in each bin is visualized on a logarithmic color scale. The encircled area corresponds to a region on 15q13 known for genetic instability, including duplications and inversions associated with highly identical blocks of flanking segmental duplications [18, 19, 21]. **b** Difference in population frequencies found in European (EUR) and non-European individuals on chromosome 17. Color intensity indicates the difference between within-population frequencies, with positive values indicating higher prevalence in the European group. Encircled is a signal that is consistent with an inversion on 17q21.31 found to be under selection in Europeans by Stefansson et al. [23]. **c** Difference in population frequencies found in the African (AFR) and South Asian (SAS) superpopulation groups on chromosome 5. Color intensity indicates the difference between within-population frequencies, with positive values indicating higher prevalence in the South Asian group

Figure 6a shows a heat map of the potential inversion alignments in chromosome 15 that were identified using the described filtering procedure. Intensity denotes the frequency of individuals within the entire 1000 Genomes phase 3 data set, shown on a logarithmic color scale. One area that stands out is a region starting around 30 Mb, with a span of template lengths between roughly 880 to 1040 kb, that shows consistently high individual frequencies reaching up to 14%. This coincides with a region on 15q13 known for genomic instability that is associated with a number of genetic disorders [18–20]. The region is characterized by complex polymorphisms including deletions and inversions, many of which are associated with highly identical blocks of flanking segmental duplications [21, 22]. The detected signal is consistent with these previously observed chromosomal rearrangements, and indicates that regions of known instability like 15q13 are possible to reproduce using the proposed filtering approach.

In addition to filtering based on alignment data, BAMSI is also designed to facilitate the handling of subsets of the 1000 Genomes data set. This allows for easy partitioning of data to perform analysis of genomic events on population or even individual level. As an example of such a use case, we consider an inversion of 17q21.31 that has been identified to have a frequency of 20% in Europeans and to be rare in other populations [23]. We extract the potential inversion alignments in chromosome 17 that come from European individuals and compare these frequencies to those of the non-European group. Figure 6b shows the difference between within-population frequencies of the European and non-European population groups, with positive values indicating higher values in the European group. Observed frequencies are overall higher in the non-European group, which could possibly be an artifact of the disproportionate sample sizes of 505 European and 2030 non-European individuals. However, an area around 43 Mb with template lengths around 600 kb stands out as having higher frequencies in Europeans. This is in line with the results of Stefansson et al. in [23] and supports the existence of an inversion in this area with higher prevalence in Europeans.

Finally, another comparison of subpopulations is shown in Fig. 6c, where the difference in frequencies between the African and South Asian population groups on chromosome 5 is shown. In this case, the majority of signals that appear with high strength have similar frequencies in both populations. A few exceptions stand out as more prevalent in either population and could be signals of e.g. ongoing selection. The filter performed was a rudimentary one, with effects of noise and alignment error likely prevalent, but the results serve to demonstrate the utility of BAMSI to gain an overview of large amounts of genomic data, detect previously known events, and to indicate areas of potential interest for further study. Genome-wide total population frequencies for the entire 1000 Genomes phase 3 data set can be found in Additional file 2.

## Discussion

A freely available deployment of BAMSI is hosted by SSC and can be accessed via http://bamsi.research.it.uu.se. As of writing, this service comprises 30 instances with 2 VCPUs, 40GB disk, 4GB RAM, and leverages the UPPMAX source of the 1000 Genomes data, along with the Amazon S3 and original FTP public mirrors, and supports download of results via HTTP. An average throughput of 452 MB/s was measured in December 2017 for 15 runs of the same query as was used for the performance testing, but with the inclusion of write to HDFS, thus giving an indication of the performance that can be achieved for a practical use scenario. As shown by the performance analysis, improvements could be gained by deploying additional workers in e.g. Amazon EC2 accessing data from S3, but this would come at an additional cost. In the current scenario, the access to a community cloud and the public mirrors allows for providing a free service with reasonable performance, illustrating the flexibility of BAMSI in adapting deployments to available infrastructure and budget. In addition to the public deployment of BAMSI, the system also contributes a more general framework for distributed processing. Compared to using complete analysis workflow systems that allow stream-based analysis on cloud platforms, e.g. Galaxy [24] and Chipster [25], the BAMSI framework is more focused on flexibility. The multi-cloud infrastructure gives flexibility in terms of resource usage, allowing for optimization of costs as well as performance. Further, BAMSI is not restricted to a predefined set of analysis tools, but possible to integrate into custom bioinformatics pipelines. We thus envision BAMSI to be a means for users with limited experience of cloud infrastructures to incorporate distributed computing into their workflows. Finally, although BAMSI is designed to work more or less out of the box, the source is open for users wishing to modify and customize it, e.g. for implementation of additional filter conditions or extension to different data sets. Currently, obtaining optimal performance from a BAMSI deployment requires evaluation of the underlying resources to configure the framework. Subsequent versions could improve on this by incorporating information on Quality-of-Service (QoS) and current infrastructure capabilities to manage the running application, e.g. by adapting worker concurrencies, task deployment and data sources dynamically. Another feature that could improve performance is adjustment of task granularity. Currently, one task comprises one BAM file, but varying task sizes could be achieved by assigning multiple files to each worker or splitting files by region to make the granularity finer. Larger tasks have the advantage of reducing communication overhead, whereas a smaller task size can increase the potential concurrency of the system and reduce the risk of unbalanced computation loads. Other scenarios where a finer granularity may be beneficial are if read failures causing tasks to be restarted are significantly affecting performance, or if pushing large files to the SR is problematic.

## Conclusions

BAMSI is intended for employment in various configurations and use-cases. The publicly available platform provides an efficient means for filtering the 1000 Genomes data, intended in particular for those without access to a private source wishing to extract small subsets of the data. More generally, BAMSI constitutes a data handling paradigm utilizing cloud services to manage large genomic data sets. As the link to any source of the data will eventually become saturated due to network limitations, the performance analysis results indicate the benefits of combining multiple resources. Further, working in cloud environments allows for post-processing in distributed computing frameworks located close to the data. An example of such a use-case is the structural variation analysis presented, in which BAMSI was used for an initial reduction of the data set, and the Hadoop framework for subsequent filtering according to a custom condition. In other scenarios, we would propose using BAMSI as a complement to existing bioinformatics workflows and tools as a pre-filtering step. With the current increase in availability of IaaS resources, our results illustrate how BAMSI provides a flexible framework with the potential to maximize the access and scientific return of large genomic data sets.

## Availability and requirements

**Project Name:** BAMSI

**Project Home Page:** http://bamsi.research.it.uu.se/

**BAMSI source:** https://github.com/NGDSG/BAMSI

**Archived version:** http://doi.org/10.5281/zenodo.1264662

**API source:** https://github.com/NGDSG/BAMSI-API

**Archived version:** http://doi.org/10.5281/zenodo.1264670

**Operating system(s):** Platform independent

**Programming language:** Python

**Other Requirements:** Deploying the framework requires Python 2.7/3.4 or later, and SAMtools 1.6 or later.

**License:** GNU General Public License v3.0

## Additional files

Additional file 1 Hive queries. The Hive queries used to filter out potential inversion alignments. (PDF 47 kb)

Additional file 2 Full-genome results. Potential inversion alignments found in all chromosomes. (PDF 7617 kb)

## Abbreviations

NGS: Next-generation sequencing
BAM/SAM: Binary/sequence alignment map
HDFS: Hadoop distributed file system
BAMSI: BAM search infrastructure
IaaS: Infrastructure as a service
SSC: SNIC science cloud
UPPMAX: Uppsala multidisciplinary center for advanced computational science
QoS: Quality-of-service
