# Supplementary material for: BAMSI: a multi-cloud service for scalable distributed filtering of massive genome data
Source: BMC Bioinformatics. 2018 Jun 26;19:240. doi: 10.1186/s12859-018-2241-z (PMC6019789; doi:10.1186/s12859-018-2241-z)
Supplement: Supplementary file 1 — Hive queries. The Hive queries used to filter out potential inversion alignments. (PDF 47 kb) [file 12859_2018_2241_MOESM1_ESM.pdf]

Hive queries

The following Hive queries were executed to perform the filtering described in section 3.2, after BAMSI had been used to perform an initial pre-filter to extract alignments with a minimum template length of 600 bp.

```
CREATE EXTERNAL TABLE all_alignments (alignment string)
location '/BAMSI_output';

CREATE TABLE chr_N(id STRING, reg STRING, chrom STRING,
Qname STRING, flag DOUBLE, pos DOUBLE,
mapq DOUBLE, cigar STRING, pnext DOUBLE,
maplen DOUBLE, tags STRING);

INSERT INTO TABLE chr_N
SELECT split(alignment, "\\s+")[0],split(alignment, "\\s+")[1],
split(alignment, "\\s+")[4] , split(alignment, "\\s+")[2] ,
cast(split(alignment, "\\s+")[3] as double),
cast(split(alignment, "\\s+")[5] as double),
cast(split(alignment, "\\s+")[6] as double ),
split(alignment, "\\s+")[7],
cast(split(alignment, "\\s+")[9] as double),
cast(split(alignment, "\\s+")[10] as double),
substr(alignment,locate(split(alignment, "\\s+")[13], alignment))
FROM all_alignments
WHERE split(alignment, "\\s+")[4] == "N";


CREATE TABLE chr_N_filtered(id STRING, reg STRING, chrom STRING,
Qname STRING, flag DOUBLE, pos DOUBLE, mapq DOUBLE, cigar STRING,
pnext DOUBLE, maplen DOUBLE, tags STRING);

INSERT INTO TABLE chr_N_filtered
SELECT * FROM chr_N
WHERE split(cigar, "M")[1] == "" AND
split(cigar, "S|M|I|D|N|H|P|=|X")[0] == split(cigar, "M")[0] AND
instr(tags, "XA") == 0;


CREATE TABLE chr_N_alignment_pairs(id STRING, reg STRING, chrom STRING,
Qname STRING, pos1 DOUBLE, pos2 DOUBLE, flag1 DOUBLE, flag2 DOUBLE,
maplen DOUBLE, mapq1 DOUBLE, mapq2 DOUBLE, cigar1 STRING, cigar2 STRING,
tags1 STRING, tags2 STRING);
```

```
INSERT INTO TABLE chr_N_alignment_pairs
SELECT t1.id, t1.reg, t1.chrom, t1.Qname, t1.pos, t2.pos, t1.flag,
t2.flag, t1.maplen, t1.mapq, t2.mapq, t1.cigar, t2.cigar, t1.tags,
t2.tags
FROM chr_N_filtered AS t1
LEFT OUTER JOIN chr_N_filtered AS t2
ON (t1.Qname = t2.Qname)
WHERE t1.maplen > 0 AND t2.maplen < 0 AND
int(t1.flag) & 16 == int(t2.flag) & 16;

CREATE TABLE bin_counts(pos1 DOUBLE, tlen DOUBLE, counter DOUBLE);

INSERT INTO TABLE bin_counts
SELECT FLOOR(pos1/1000), FLOOR(maplen/1000), count(distinct(id))
from chr_N_alignment_pairs group by FLOOR(pos1/1000), FLOOR(maplen/1000);

INSERT OVERWRITE LOCAL DIRECTORY "/results/chrN"
ROW FORMAT DELIMITED
FIELDS TERMINATED BY ','
SELECT FLOOR(pos1/1000000), FLOOR(maplen/10000), count(distinct(id))
FROM chr_N_alignment_pairs AS a
WHERE EXISTS(SELECT pos1, tlen FROM bin_counts AS p
WHERE p.pos1 == FLOOR(a.pos1/1000) AND
p.tlen == FLOOR(a.maplen/1000) AND counter >= 20)
GROUP BY FLOOR(pos1/1000000), FLOOR(maplen/10000);
```
