# Supplementary material for: BAMSI: a multi-cloud service for scalable distributed filtering of massive genome data
Source: BMC Bioinformatics. 2018 Jun 26;19:240. doi: 10.1186/s12859-018-2241-z (PMC6019789; doi:10.1186/s12859-018-2241-z)
Supplement: Supplementary file 2 — Full-genome results. Potential inversion alignments found in all chromosomes. (PDF 7617 kb) [file 12859_2018_2241_MOESM2_ESM.pdf]

Full-genome results

Results of the structural variation analysis described in section 3.2, for all chromosomes.

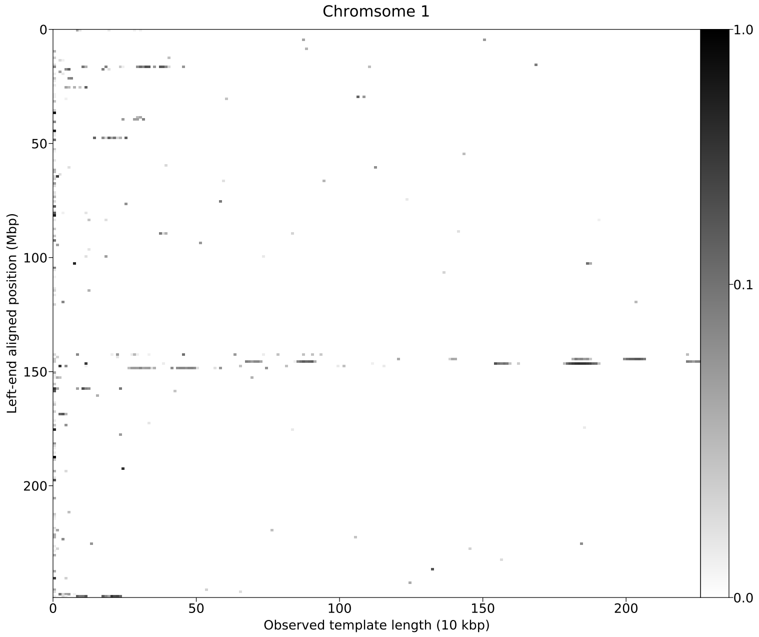

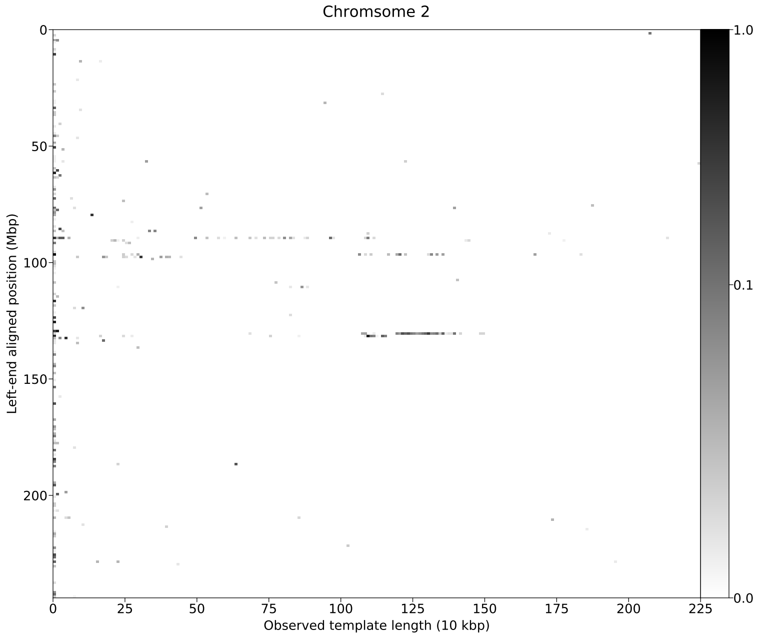

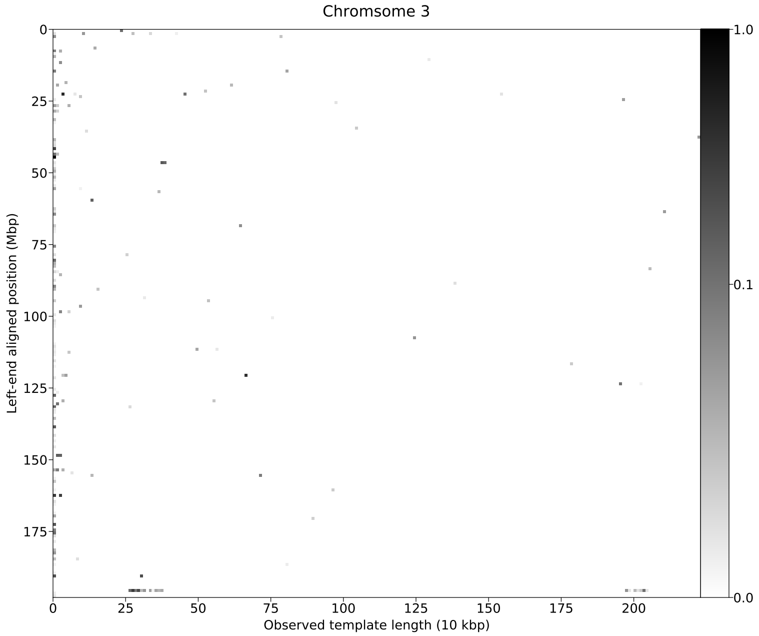

Chromsome 3
Left-end aligned position (Mbp)
Observed template length (10 kbp)
0
25
50
75
100
125
150
175
200
1.0
0.1
0.0

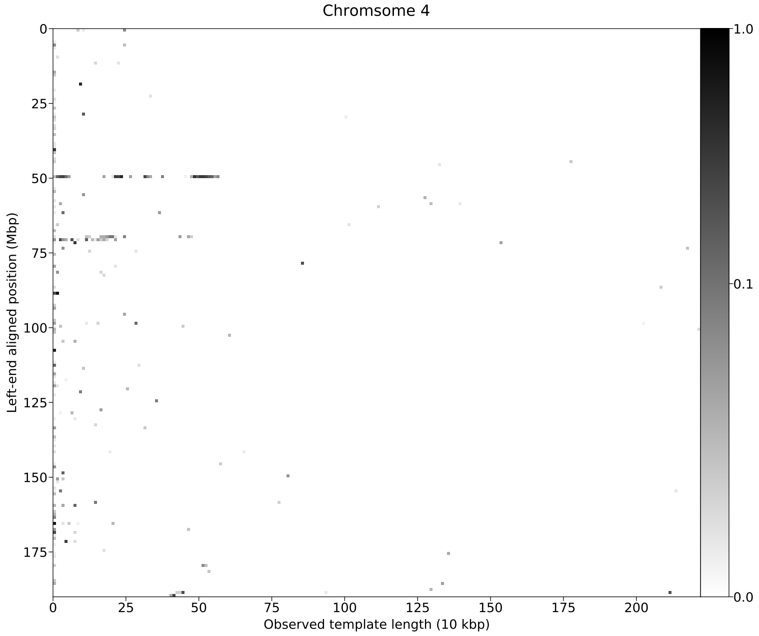

Chromsome 4
Left-end aligned position (Mbp)
Observed template length (10 kbp)
0
25
50
75
100
125
150
175
200
1.0
0.1
0.0

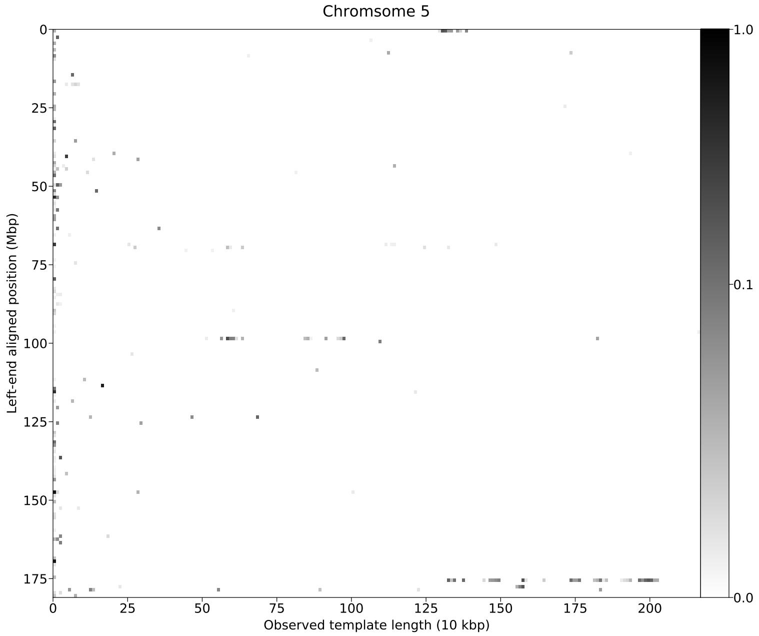
Chromsome 5
Left-end aligned position (Mbp)
Observed template length (10 kbp)
0
25
50
75
100
125
150
175
200
1.0
0.1
0.0

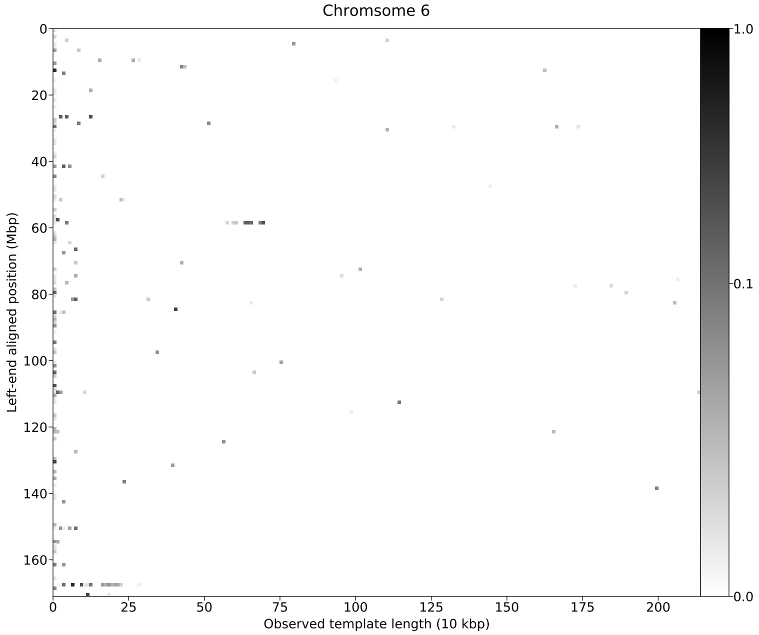
Chromsome 6
Left-end aligned position (Mbp)
Observed template length (10 kbp)
0
20
40
60
80
100
120
140
160
25
50
75
125
150
175
200
1.0
0.1
0.0

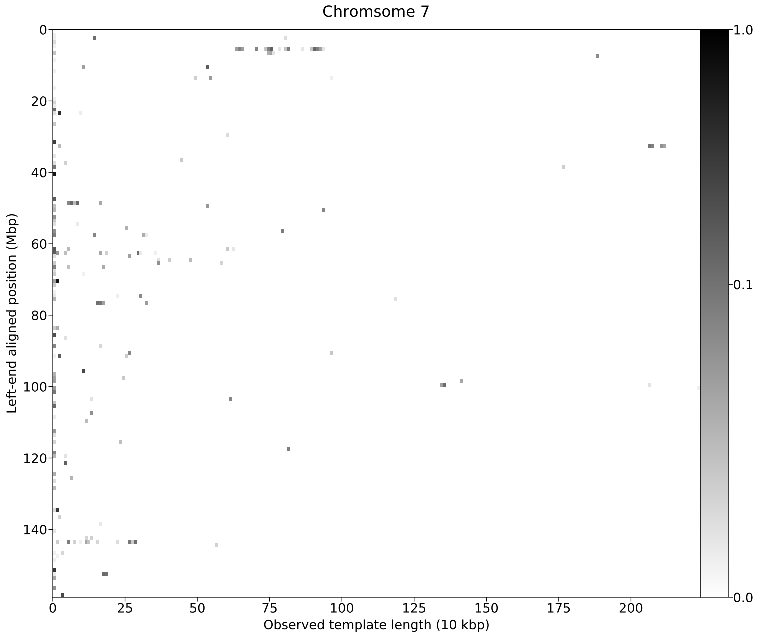
Chromsome 7
Left-end aligned position (Mbp)
Observed template length (10 kbp)
0
20
40
60
80
100
120
140
0
25
50
75
100
125
150
175
200
1.0
0.1
0.0

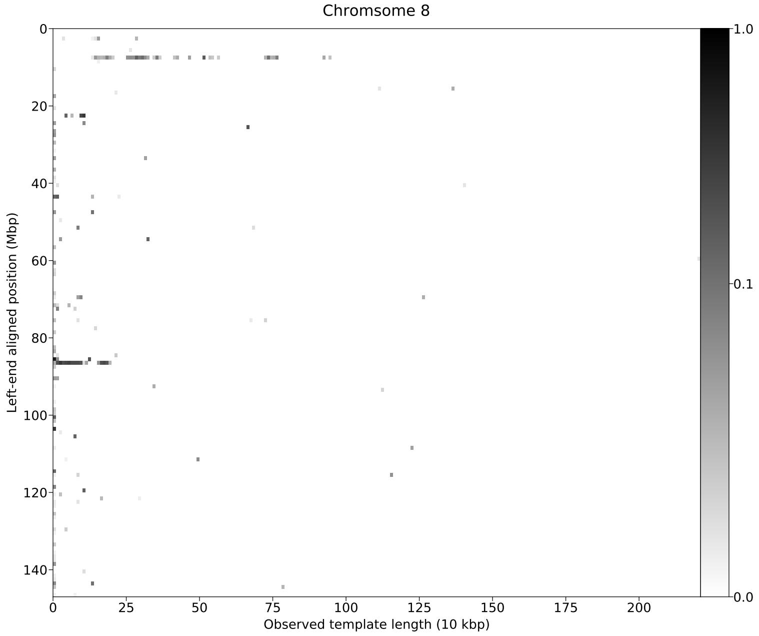
Chromsome 8
Left-end aligned position (Mbp)
Observed template length (10 kbp)
0
20
40
60
80
100
120
140
0
25
50
75
100
125
150
175
200
1.0
0.1
0.0

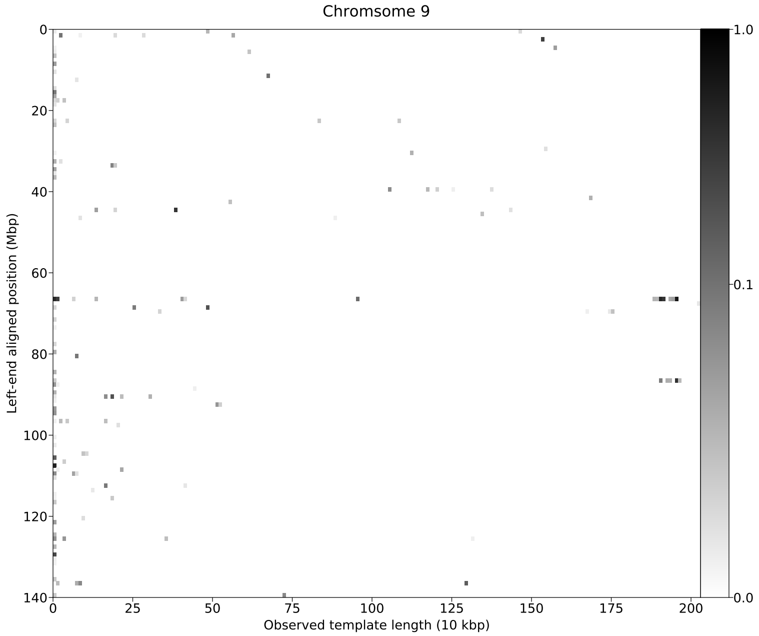

Chromsome 9
Left-end aligned position (Mbp)
Observed template length (10 kbp)
0
20
40
60
80
100
120
140
0
25
50
75
100
125
150
175
200
1.0
0.1
0.0

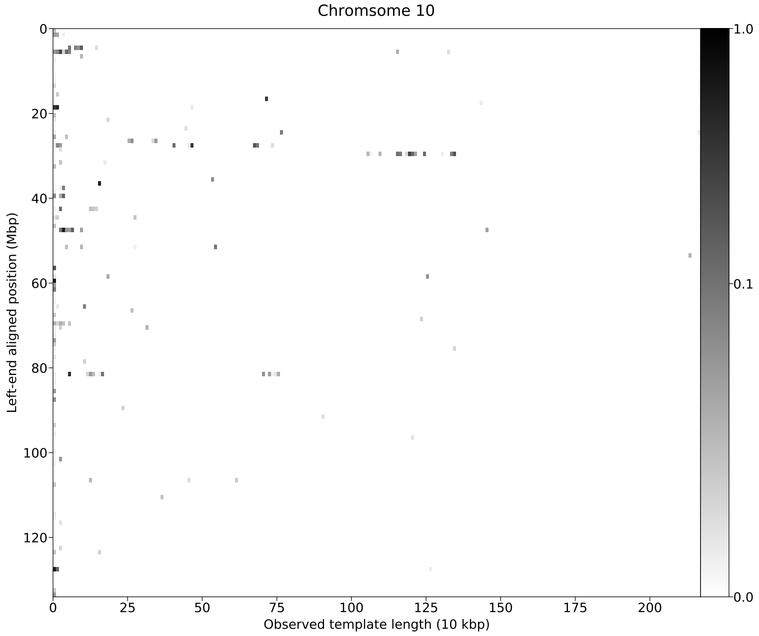

Chromsome 10
Left-end aligned position (Mbp)
Observed template length (10 kbp)
0
20
40
60
80
100
120
0
25
50
75
100
125
150
175
200
1.0
0.1
0.0

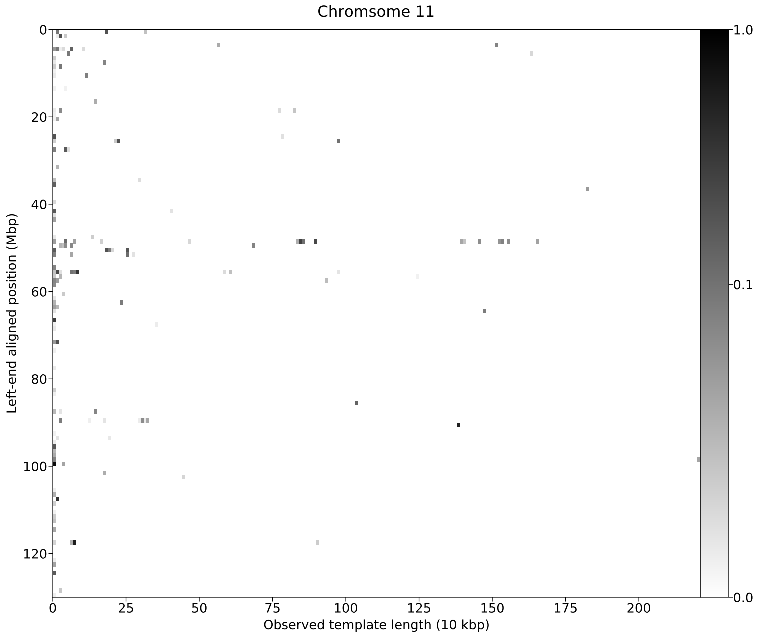
Chromsome 11
Left-end aligned position (Mbp)
Observed template length (10 kbp)
0
20
40
60
80
100
120
0
25
50
75
100
125
150
175
200
1.0
0.1
0.0

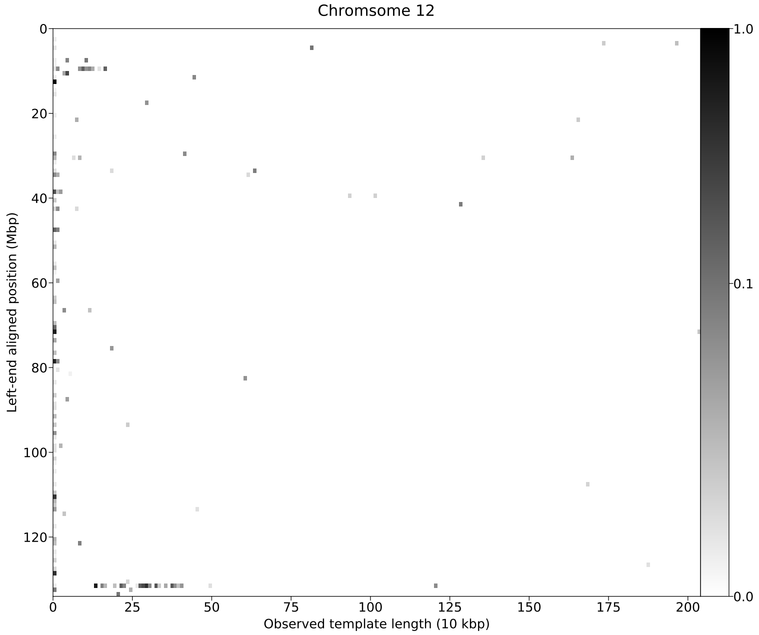
Chromsome 12
Left-end aligned position (Mbp)
Observed template length (10 kbp)
0
20
40
60
80
100
120
0
25
50
75
100
125
150
175
200
1.0
0.1
0.0

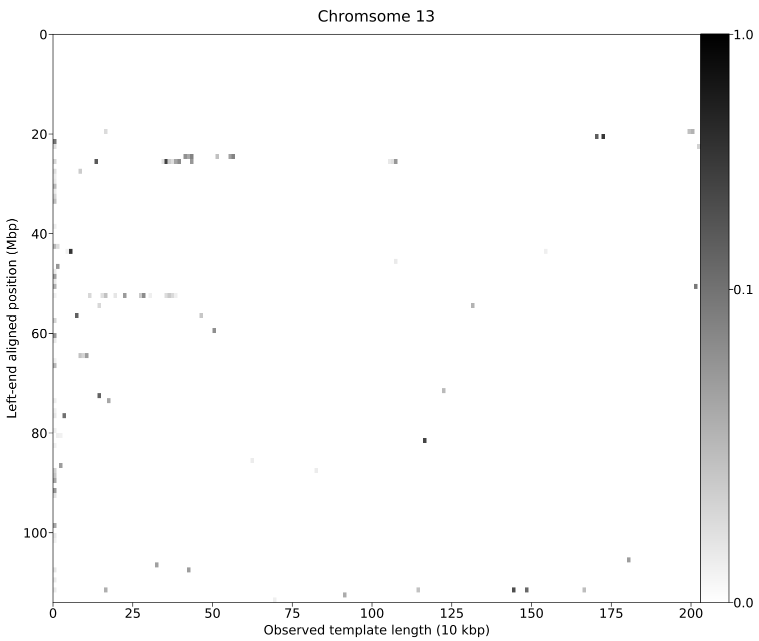

Chromsome 13
Left-end aligned position (Mbp)
Observed template length (10 kbp)
0
20
40
60
80
100
0
25
50
75
100
125
150
175
200
1.0
0.1
0.0

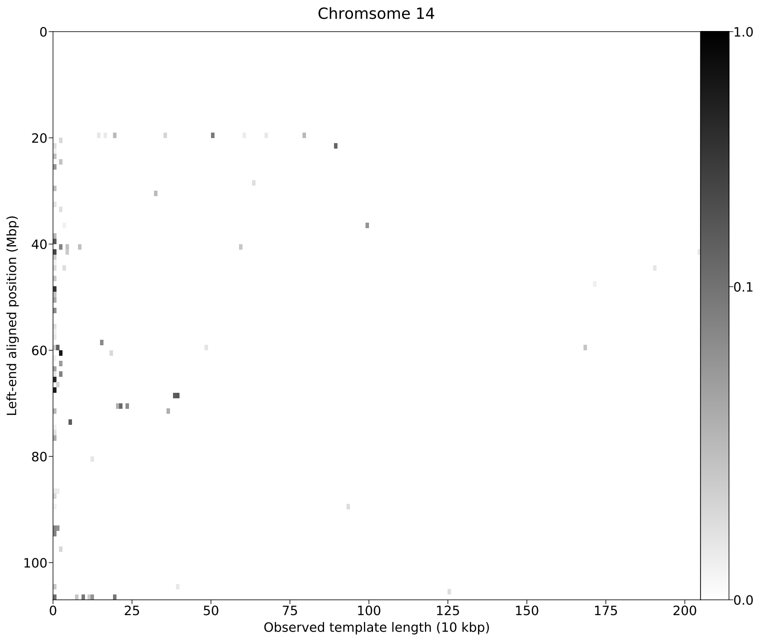

Chromsome 14
Left-end aligned position (Mbp)
Observed template length (10 kbp)
0
20
40
60
80
100
0
25
50
75
100
125
150
175
200
1.0
0.1
0.0

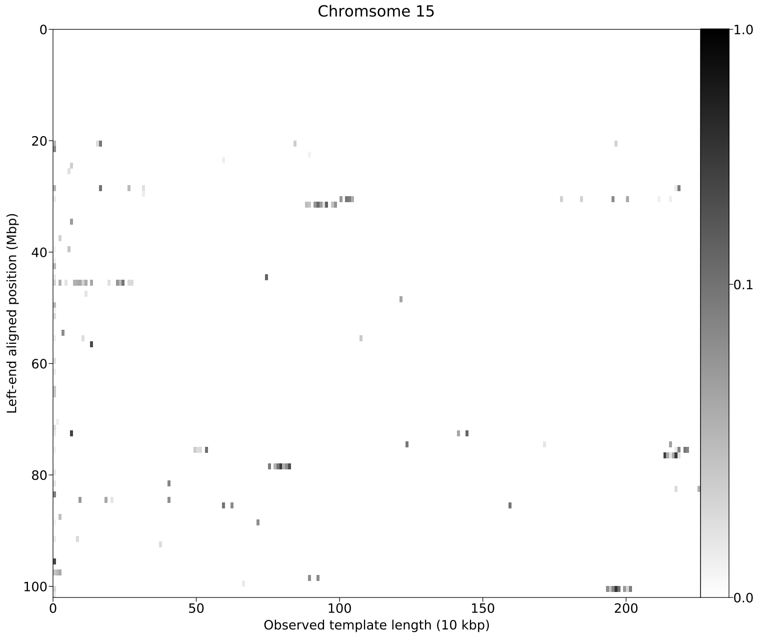

Chromsome 15
Left-end aligned position (Mbp)
Observed template length (10 kbp)
0
20
40
60
80
100
0
50
100
150
200
1.0
0.1
0.0

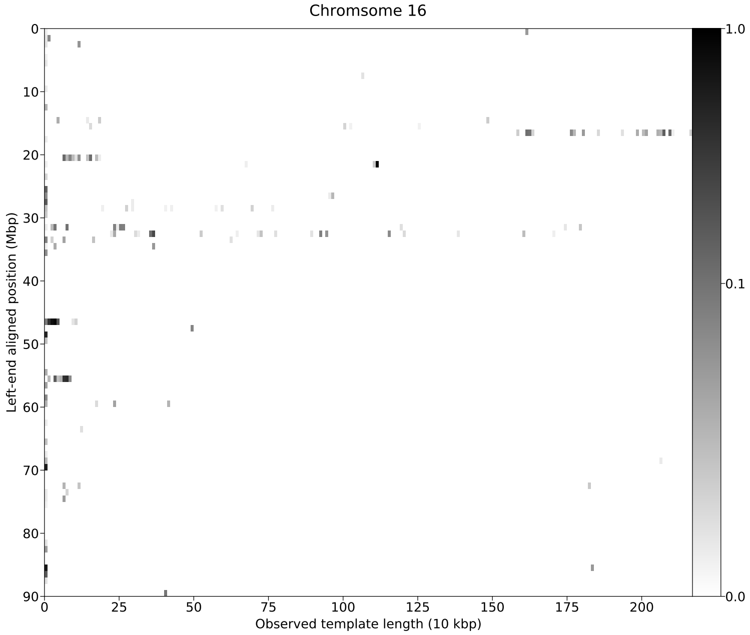

Chromsome 16
Left-end aligned position (Mbp)
Observed template length (10 kbp)
0
10
20
30
40
50
60
70
80
90
0
25
50
75
100
125
150
175
200
1.0
0.1
0.0

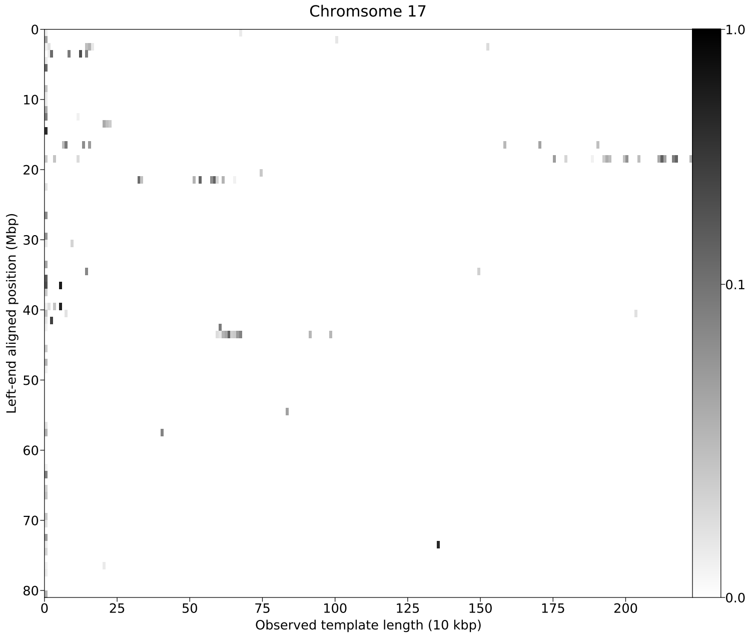
Chromsome 17
Left-end aligned position (Mbp)
Observed template length (10 kbp)
0
10
20
30
40
50
60
70
80
0
25
50
75
100
125
150
175
200
1.0
0.1
0.0

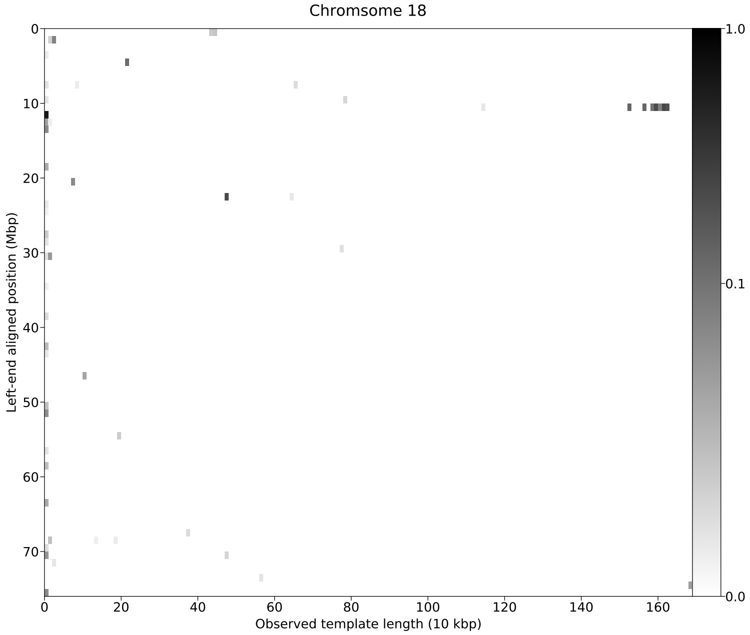
Chromsome 18
Left-end aligned position (Mbp)
Observed template length (10 kbp)
0
10
20
30
40
50
60
70
0
20
40
60
80
100
120
140
160
1.0
0.1
0.0

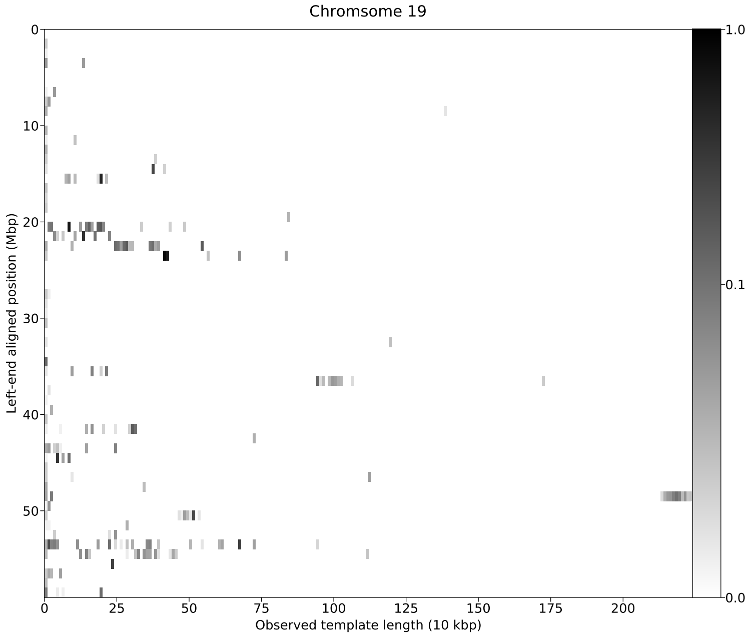
Chromsome 19
Left-end aligned position (Mbp)
Observed template length (10 kbp)
0
10
20
30
40
50
25
75
100
125
150
175
200
1.0
0.1
0.0

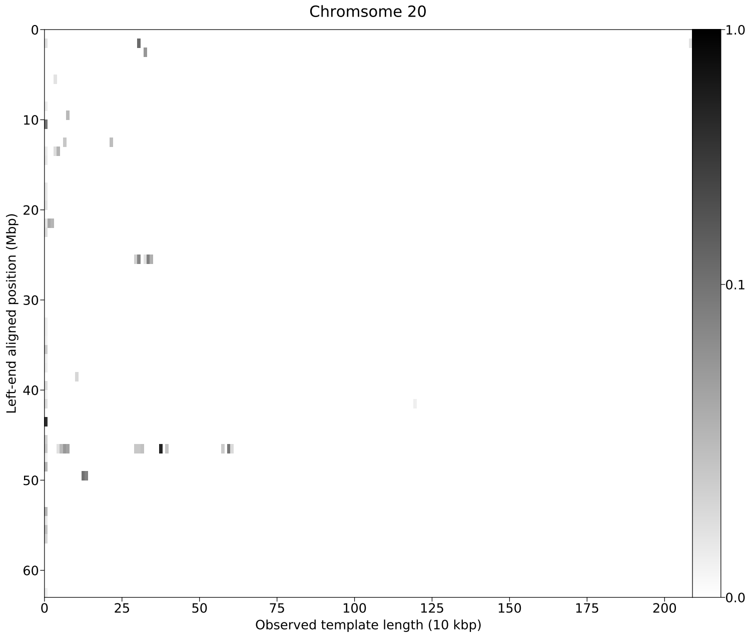
Chromsome 20
Left-end aligned position (Mbp)
Observed template length (10 kbp)
0
10
20
30
40
50
60
25
75
100
125
150
175
200
1.0
0.1
0.0

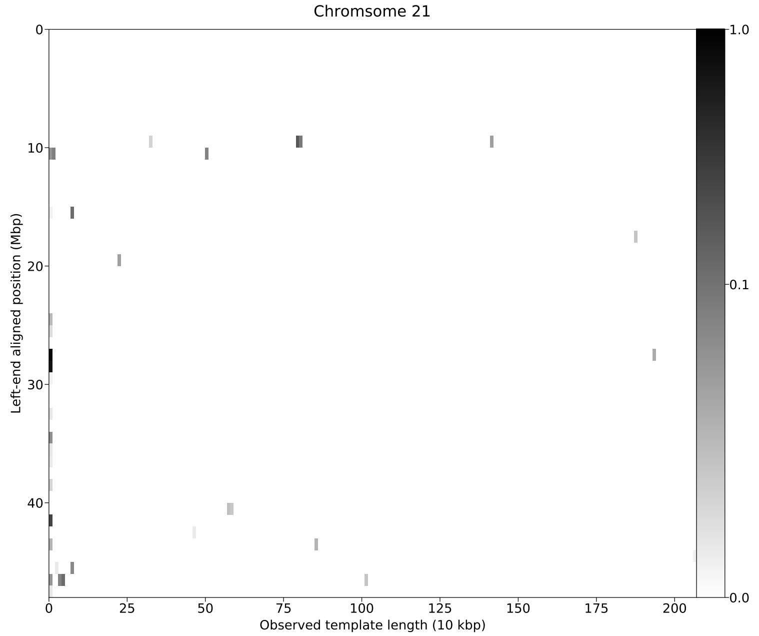
Chromsome 21
Left-end aligned position (Mbp)
Observed template length (10 kbp)
0
10
20
30
40
25
50
75
100
125
150
175
200
1.0
0.1
0.0

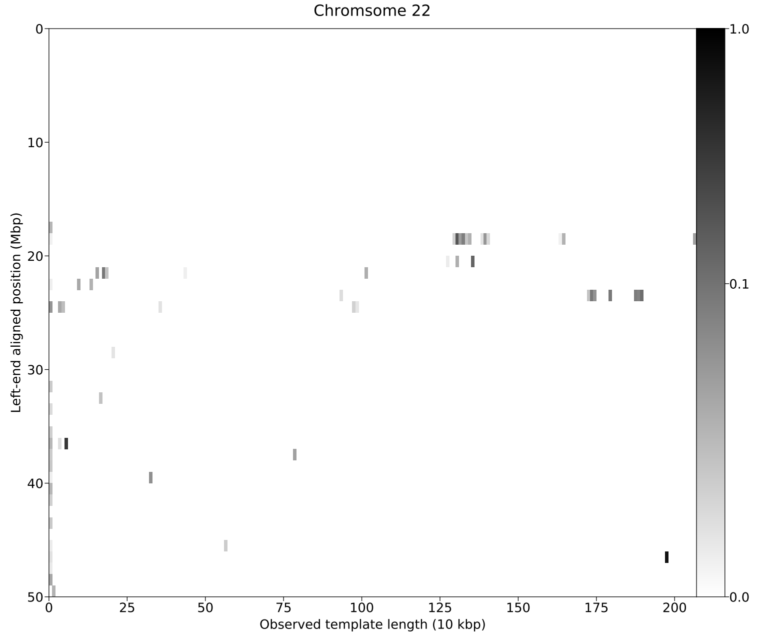
Chromsome 22
Left-end aligned position (Mbp)
Observed template length (10 kbp)
0
10
20
30
40
50
25
75
100
125
150
175
200
1.0
0.1
0.0

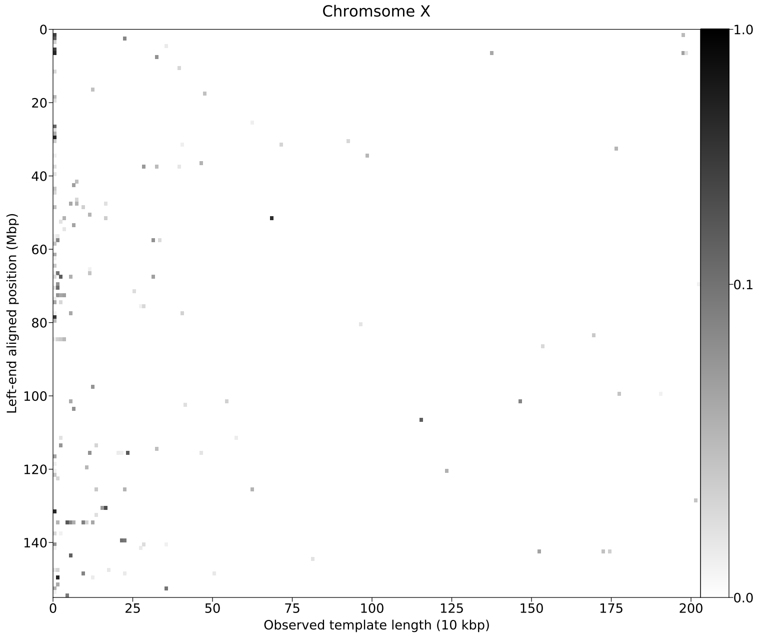
Chromsome X
Left-end aligned position (Mbp)
Observed template length (10 kbp)
0
20
40
60
80
100
120
140
0
25
50
75
100
125
150
175
200
1.0
0.1
0.0

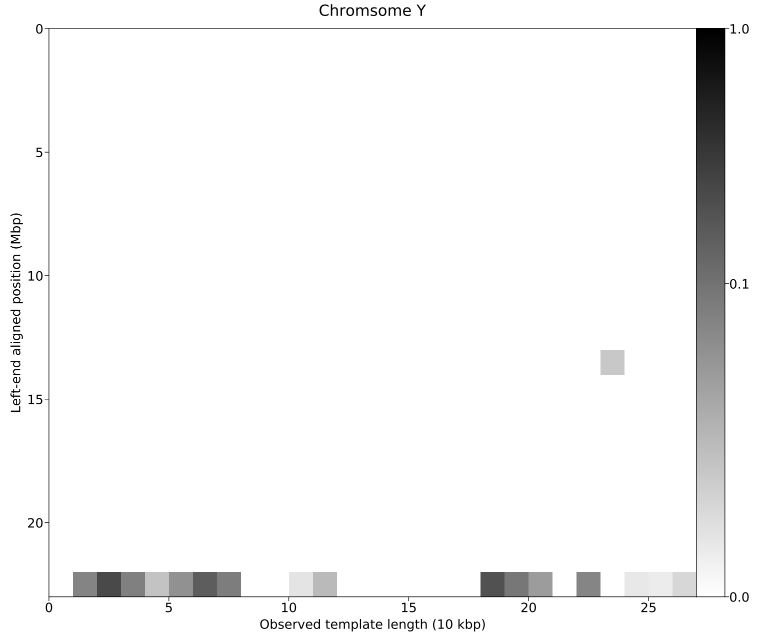
Chromsome Y
Left-end aligned position (Mbp)
Observed template length (10 kbp)
0
5
10
15
20
0
5
10
15
20
25
1.0
0.1
0.0
